# Symptom and problem clusters in German specialist palliative home care - a factor analysis of non-oncological and oncological patients’ symptom burden

**DOI:** 10.1186/s12904-023-01296-0

**Published:** 2023-11-17

**Authors:** Daniela Gesell, Farina Hodiamont, Julia Wikert, Eva Lehmann-Emele, Claudia Bausewein, Friedemann Nauck, Maximiliane Jansky, , for the COMPANION study group

**Affiliations:** 1 Department of Palliative Medicine, LMU University Hospital, LMU Munich, Munich, Germany; 2 Department of Palliative Medicine, University Medical Center Goettingen, Goettingen, Germany

**Keywords:** Specialist palliative home care, Symptom cluster, Complexity, Exploratory factor analysis, Confirmatory factor analysis, End-of-life care

## Abstract

**Background:**

Specialist palliative home care (SPHC) aims to maintain and improve patients’ quality of life in the community setting. Symptom burden may differ between oncological and non-oncological patients. However, little is known about diagnosis-related differences of SPHC patients. This study aims to describe the prevalence of physical symptom burden and psychosocial problems of adult patients in SPHC, and to evaluate diagnosis-related symptom clusters.

**Methods:**

Secondary analysis of data from a prospective, cross-sectional, multi-centre study on complexity of patients, registered at the German Register for Clinical Studies (DRKS trial registration number: DRKS00020517, 12/10/2020). Descriptive statistics on physical symptom burden and psychosocial problems at the beginning of care episodes. Exploratory and confirmatory factor analyses to identify symptom and problem clusters.

**Results:**

Seven hundred seventy-eight episodes from nine SPHC teams were included, average age was 75 years, mean duration of episode 18.6 days (SD 19.4). 212/778 (27.2%) had a non-oncological diagnosis. Main burden in non-oncological episodes was due to poor mobility (194/211; 91.9%) with significant diagnosis-related differences (χ² = 8.145, df = 1, *p* = .004; oncological: 472/562; 84.0%), and due to weakness (522/565; 92.4%) in oncological episodes. Two symptom clusters (psychosocial and physical) for non-oncological and three clusters (psychosocial, physical and communicational/practical) for oncological groups were identified. More patients in the non-oncological group compared to the oncological group showed at least one symptom cluster (83/212; 39.2% vs. 172/566; 30.4%).

**Conclusion:**

Patients with non-oncological diseases had shorter episode durations and were more affected by symptom clusters, whereas patients with oncological diseases showed an additional communicational/practical cluster. Our findings indicate the high relevance of care planning as an important part of SPHC to facilitate anticipatory symptom control in both groups.

**Supplementary Information:**

The online version contains supplementary material available at 10.1186/s12904-023-01296-0.

## Background

Most patients with life limiting diseases suffer from multiple co-occurring symptoms, like pain, fatigue, insomnia and depression, [1, 2] with a potentially negative impact on functional and cognitive status, [3] and quality of life  [4, 5]. Often, these symptoms and problems don’t occur isolated but in groups or symptom clusters which are defined as two or more concurrent symptoms in stable groups, distinct from other clusters [2, 6]. The study of the prevalence and stability of symptom clusters is clinically essential to develop intervention strategies [7] and to maintain and improve patients’ quality of life, as compared to single symptoms, symptom clusters worsen patients’ outcomes [2].

Current studies focusing on symptom clusters of oncological patient groups with different life-limiting illness, [4, 5, 7–11] identified multiple symptom clusters, like anxiety-depression, nausea-vomiting, nausea-appetite loss and fatigue- dyspnea-drowsiness-pain [12]. Some studies evaluated symptom clusters in patients with advanced non-oncological diseases and also identified various symptom clusters, e.g. a study on patients with chronic kidney disease found two clusters: first, weakness, mouth problems, poor mobility, difficulty sleeping, feeling anxious, and feeling depressed, and second, nausea, vomiting, and diarrhoea [13]. Another United Kingdom multicentre, cross-sectional study developed a symptom cluster model for patients with chronic obstructive pulmonary disease and identified three clusters, one respiratory related, one psychological and one cough-insomnia related symptom cluster [14]. A German study on symptom clusters in inpatient palliative care settings found no significant differences, with five clusters for oncological and non-oncological patients, respectively. One cluster identified was nausea and vomiting, another was anxiety, tension and depression and slight variations in the distribution of the other clusters in symptoms and problems like weakness, tiredness, loss of appetite, and assistance with activities of daily living [15]. Possibly, the pattern and progression of symptoms differ within diagnosis groups with different stages of disease [16].

While most studies focused on inpatient specialist palliative care (SPC) settings, a substantial number of patients receive SPC in the home care setting [17]. Therefore, the focus of the present study is on patients who were cared for by a specialist palliative home care (SPHC) team. To preserve patients’ autonomy and quality of life at the end of life through comprehensive support in pain management and symptom control is an important responsibility of SPHC teams [18]. Pro-active symptom management is especially important in community based palliative care, as the care system can be easily destabilized in the home care setting [19], which may result in unnecessary hospital admissions.

Emerging evidence suggests that very diverse symptom clusters occur in patients with a variety of chronic, oncological and non-oncological diagnoses, [2] however, little is known about diagnosis-related differences in the prevalence of symptoms and problems, and the occurring symptom clusters of patients receiving SPHC. Identifying symptom clusters can support care planning, improve quality of care and facilitate better outcomes [2]. Therefore, the aim of this study was to describe the prevalence of physical symptom burden and psychosocial problems of adult patients in SPHC, and to evaluate diagnosis-related symptom clusters.

## Materials and methods

The reporting of this study follows the ‘Strengthening the Reporting of Observational Studies in Epidemiology’ (STROBE) guideline and in addition the German version of ‘STandardized Reporting Of Secondary data Analyses’ (STROSA) recommendation [20, 21].

### Study design

This study is a secondary analysis of data collected in COMPANION [22], a project to develop a case-mix classification for adult palliative care patients in Germany, funded by the Innovations Fund of the Federal Joint Committee (grant number 01VSF18018). The study is registered at the German Register for Clinical Studies (DRKS trial registration number: DRKS00020517). Details on the study are reported elsewhere [22].

### Setting and population

From 04/21 to 09/22, nine SPHC teams consecutively collected data on all newly admitted patients. Data was collected during the whole episode of care, defined as the time between admission and discharge, change of location, or death. Data was anonymously collected by the professionals of the participating SPHC teams, thereby all patients (age ≥ 18 years) could be included irrespective of their condition and ability to consent. Data quality was maximized by training the multidisciplinary SPHC staff in assessments during data collection, reducing missing data and data bias. Regular feedback on the plausibility and accuracy of the documented assessments was provided over a three month period [22].

### Data sources and measures

Each team collected sociodemographic data, functional status and data on patients’ needs reflecting complexity over a three months period. Sociodemographic data was documented once at the beginning of each episode of care (age in years, gender and main diagnosis) [22]. Length of episode of care was automatically calculated as the difference in days between date of admission and date of discharge or death. The Australia-modified Karnofsky Performance Status (AKPS) was recorded to describe the functional status of the patient [23]. The Integrated Palliative care Outcome Scale (IPOS) was used to reflect patients’ symptom burden, psychosocial burden, concerns of relatives and practical problems with 17 items on a 5-point Likert scale. Responses to IPOS range from “0” (no physical or psychosocial burden of patients and/or their relatives) to “4” (overwhelming physical or psychosocial burden/no psychosocial problems and needs addressed) [24]. This instrument was developed specifically for patients in palliative care and reflects all dimensions of SPC, separated into three subscales: physical symptoms, emotional symptoms and communication/practical issues. There are two validated versions available, one for patients’ self-report and one proxy version for professionals, which we used in this study. Both versions demonstrated similar results with acceptable to good agreement [24]. The main difference between the proxy and self-reported version is the existence of the ‘cannot assess’ option in the proxy version, as additional answer for professionals if the symptom or problem is not assessable. For each episode, we included the highest assessed value of the day of admission or first day of care episode, if there were multiple assessments on the first day.

### Methods of analysis

Sociodemographic characteristics of each included episode of care were descriptively analyzed. Prevalence of symptoms and problems was defined as any IPOS item assessed at least with a value of ‘2’, as in other studies [14]. Chi-Square tests and t-tests were calculated with Bonferroni-adjusted *p*-values < 0.05 considered to be statistically significant. Descriptive analyses were conducted using IBM SPSS statistics 26.

To evaluate symptom clusters, exploratory factor analyses (EFA) and confirmatory factor analyses (CFA) were performed: one for patients with oncological and one for patients with non-oncological diagnosis. All patients with ICD-10 Codes C01- C92.90, D03.4, D37.6, D43.2, D46.9, D47.4, D48 and D48.2 were defined as oncological, other patients as non-oncological (Supplementary Table 1).

#### Exploratory factor analysis

For the identification of symptom clusters, physical symptom burden and psychosocial problems from IPOS, were included for EFA, using IBM SPSS statistics 26. EFA assumes that the symptoms in each cluster are correlated by a latent factor [25]. Factor analysis is used to predict a set of latent factors responsible for the covariance between a set of symptoms and assumes that the symptoms occur in a cluster linked by latent factor. Symptoms attributable to this latent factor would have stronger association with each other than with symptoms influenced by another latent factor [16, 26]. As an empirically derived value, IPOS items with a prevalence greater than 20% were included in the analysis [14]. Complete case analyses were conducted. IPOS items that could not be assessed (‘cannot assess’) were treated as missing values. Principal axis analysis method and varimax rotation were used to extract relevant factors [27]. Kaiser-Meyer-Olkin (KMO) values > .50 and the significance of Bartlett’s test of Sphericity were used to verify the factorability of the data [28]. The interpretation of the factors was performed a posteriori [29]. The number of factors was determined by extracting only factors with eigenvalues > 1.0. To simplify interpretation of clustering, symptoms with factor loading > .50 were considered to load on a factor.

#### Confirmatory factor analysis

As part of a structural equation modelling, we used CFA to establish structural validity of the identified factors, using IBM SPSS AMOS 29. By this application, standardized multivariate analysis methods such as regression, factor analysis, correlation, and analysis of variance of IBM SPSS statistics could be extended. We performed a maximum likelihood estimation [24]. A relatively good fit of the factor solutions from the EFA and the observed data can be assumed using chi-square, confirmatory fit index (CFI, value > .95), root mean square error of approximation (RMSEA, value < .06) and standardized root mean squared residual (SRMR, value < .08) [30]. It is common to use the chi-square value as a descriptive value and to set it in relation to the degrees of freedom with a ratio of less than 2.5 indicating a good model fit [29].

## Results

### Participants and descriptive data

Seven hundred seventy-eight care episodes were included after removing 17 episodes with missing diagnosis. There were 212 (27.2%) episodes from patients with non-oncological diagnosis and 566 (72.8%) episodes from oncological (Table 1) with no significant differences regarding gender. Age was between 23 and 102 years with patients in the non-oncological groups being older. Median AKPS of non-oncological was 20% and of oncological episodes 40%. Mean duration of a care episode was 4.5 days longer in the oncological than in the non-oncological group. Significantly more episodes of non-oncological patients ended with death, while more than half of oncological episodes ended with discharge (e.g. stabilization or admission to hospital).

**Table 1:** Clinical characteristics per episode (*n* = 778)

Characteristics	Total	Non-oncological	Oncological	*p*-value
Total (%)	778	212 (27.2)	566 (72.8)	
Age, years *mean ± SD*	75.3 ± 12.2	81.6 ± 10.5	72.9 ± 11.9	< .001
Gender, *n (%)*				.055
Female	404 (51.9)	122 (57.5)	282 (49.8)	
Male	374 (48.1)	90 (42.5)	284 (50.2)	
AKPS, *median (range)*	40 (10–90)	20 (10–70)	40 (10–90)	
Length of episode, mean ± SD, days	18.6 ± 19.4	15.3 ± 17.1	19.8 ± 20.0	.004
Episodes end with death, *n (%)*	400 (51.4)	141 (66.5)	259 (45.8)	.000
missing *n (%)*	4 (0.5)	0	4 (0.7)	
Diagnosis, *n (%)*				
Oncological			566 (100.0)	
Lip, mouth, pharynx			12 (2.1)	
Gastrointestinal			158 (27.9)	
Respiratory			130 (23.0)	
Melanoma			12 (2.1)	
Neurology			9 (1.6)	
Mamma			51 (9.0)	
Genitourinary			123 (21.7)	
Eye, Head, CNS			10 (1.8)	
Endocrinology			1 (0.2)	
Others			60 (10.6)	
Non-oncological		212 (100.0)		
Mental and behavioral disorders		30 (14.2)		
Neurology		37 (17.5)		
Circulatory		71 (33.5)		
Respiratory		38 (17.9)		
Gastrointestinal		18 (8.5)		
Genitourinary		16 (7.5)		
Others		2 (0.9)		

### Prevalence of symptom burden and psychosocial problems

In almost all episodes of patients with non-oncological (210/212, 99.1%) and with oncological (563/566, 99.5%) diagnoses, at least one symptom or problem, in 181/212 (85.4%) and 495/566 (87.5%) episodes ≥ 5 symptoms or problems were prevalent. In 86/212 (40.6%) non-oncological and in 204/566 (36.0%) oncological episodes, patients were affected by at least five severe or overwhelming symptoms or problems (Supplementary Table 2/3). The most prevalent symptom burden were weakness and poor mobility in episodes of both groups, with significantly higher prevalence of poor mobility in non-oncological group (χ² = 8.145, df = 1, *p* = .004) (Table 2). Gastrointestinal symptoms such as nausea, vomiting and constipation had the lowest prevalence, with significantly higher prevalence in nausea in the oncological group (χ² = 7.213, df = 1, *p* = .007).

**Table 2:** Prevalence of symptom burden and psychosocial problems^a^

IPOS items	Non-Oncological		Oncological		Chi-square	*p*-value
	Prevalence		Prevalence			
pain	98/212	46.2%	324/566	57.2%	7.543	.006
shortness of breath	84/210	40.0%	165/566	29.2%	8.272	.004
weakness or lack of energy	186/210	88.6%	522/565	92.4%	2.826	.093
nausea	27/212	12.7%	120/566	21.2%	7.213	.007
vomiting	16/212	7.5%	62/566	11.0%	1.985	.159
poor appetite	118/212	55.7%	378/566	66.8%	8.259	.004
constipation	56/212	26.4%	160/565	28.3%	0.278	.598
sore or dry mouth	70/208	33.7%	170/565	30.1%	0.903	.342
drowsiness	118/210	56.2%	279/563	49.6%	2.695	.101
poor mobility	194/211	91.9%	472/562	84.0%	8.145	.004
patient anxiety	74/211	35.1%	346/561	61.7%	43.750	.000
family anxiety	156/211	73.9%	414/563	73.5%	0.013	.911
depression	72/207	34.8%	321/557	57.6%	31.538	.000
feeling at peace	50/209	23.9%	232/558	41.6%	20.382	.000
sharing feelings	50/206	24.3%	184/558	33.0%	5.363	.021
information	33/207	15.9%	123/558	22.0%	3.462	.063
practical matters	65/204	31.9%	226/548	41.2%	5.511	.019

^a^IPOS values of 0-1 of physical and/or psychosocial burden defined ‘not prevalent’ and IPOS values of 2-4 defined ‘prevalent’

### Symptom/problem clusters of non-oncological patients

In the EFA, 64/212 episodes from non-oncological patients and 14 variables of IPOS with prevalence > 20% were included (Table 3). Four factors had an eigenvalue greater than 1. After considering the screeplot (Supplementary Fig. 1) and contextual review, a two-factor solution was chosen as factors 3 and 4 had small eigenvalues and consisted of only one variable each. Bartlett’s test of sphericity was significant (χ² = 347.99, df = 91, *p* < .001), KMO was 0.74, indicating middling suitability of the correlation matrix for EFA. The first factor included patient anxiety, family anxiety, depression and feeling at peace. The second factor included weakness, poor appetite, drowsiness and poor mobility. The rotated loadings of the factors are shown in Table 3.

**Table 3:** Factor loadings of EFA

	*Non-oncological (n = 64)*		*Oncological (n = 325)*		
**IPOS items**	*Factor 1*	*Factor 2*	*Factor 1*	*Factor 2*	*Factor 3*
pain	0.205	0.229	0.266	0.139	0.006
shortness of breath	0.006	0.136	0.098	0.220	-0.030
weakness or lack of energy	0.324	**0.714**	0.168	**0.725**	0.133
nausea	**-**	**-**	0.099	0.315	-0.021
vomiting	**-**	**-**	**-**	**-**	**-**
poor appetite	0.188	**0.593**	0.181	**0.556**	0.022
constipation	0.095	0.436	0.198	0.208	0.104
sore or dry mouth	-0.202	0.417	-0.015	**0.495**	0.019
drowsiness	-0.131	**0.751**	-0.049	**0.479**	0.039
poor mobility	0.386	**0.547**	0.092	**0.524**	0.001
patient anxiety	**0.834**	0.116	**0.950**	0.093	0.063
family anxiety	**0.789**	0.162	**0.594**	0.131	0.291
depression	**0.934**	0.137	**0.798**	0.100	0.186
feeling at peace	**0.734**	0.038	**0.583**	0.063	0.352
sharing feelings	0.335	-0.146	0.059	-0.070	**0.703**
information	**-**	**-**	0.156	0.035	**0.602**
practical matters	0.267	0.130	0.296	0.095	**0.494**
Kaiser-Meyer-Olkin	0.74		0.76		
Bartlett sphericity Test	< 0.001		< 0.001		
% of variance	23.65	16.13	15.93	11.56	8.63

**Fig. 1:**
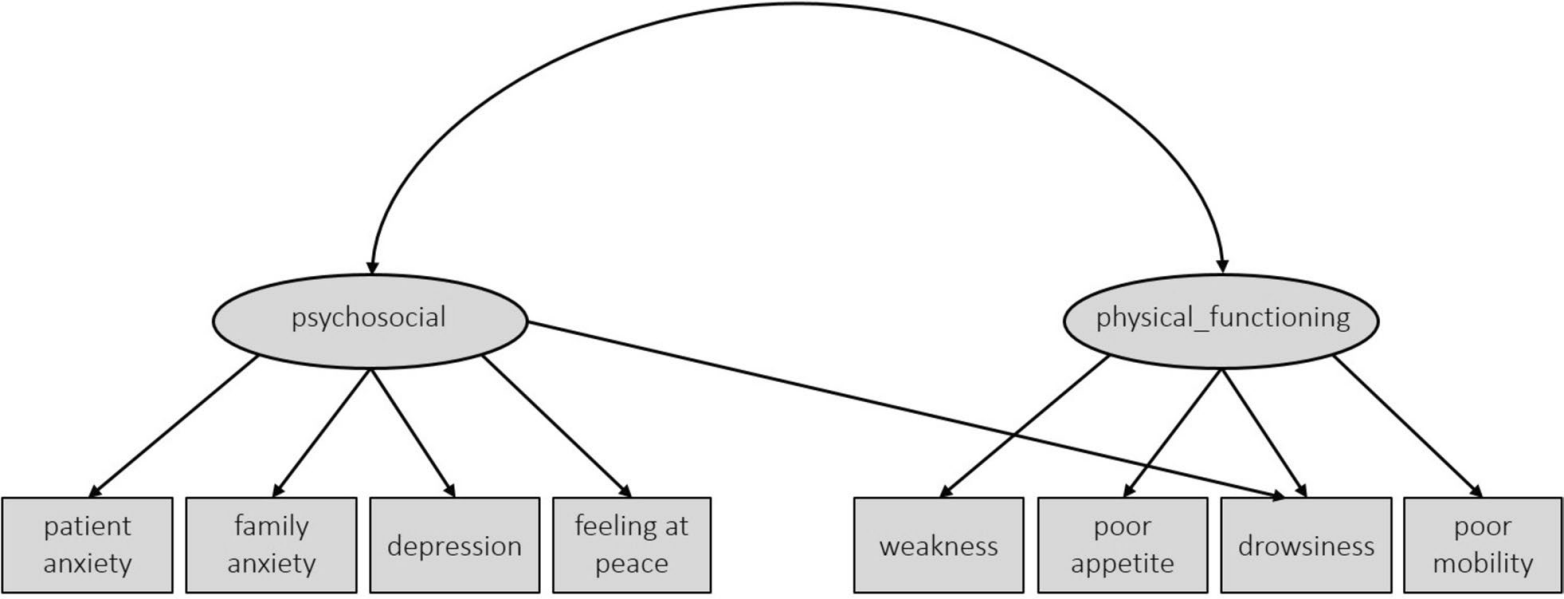
Factor model of CFA for non-oncological episodes

We conducted CFA to verify the two-factor model. First, the results of CFA identified that the designated factor model did not have a good fit (χ² = 29.66, df = 19, *p* = .056, CFI = .958, SRMR = .0948, RMSEA = .09). We identified a cross loading of ‘drowsiness’ and modified the factor model. Consequently, the final fit indicated an adequate model fit (χ² = 22.38, df = 18, *p* = .216, CFI = .983, SRMR = .0586, RMSEA = .06). Based on this the following symptom clusters were designated: A psychosocial cluster (patient anxiety, family anxiety, depression, feeling at peace and drowsiness) and a physical functioning related cluster (weakness, poor appetite, drowsiness and poor mobility) (Fig. 1).

### Symptom/problem clusters of oncological patients

In the EFA, 325/566 episodes with oncological patients and 16 variables of IPOS with prevalence > 20% could be included (Table 3). Five factors had an eigenvalue greater than 1. The screeplot (Supplementary Fig. 2) suggested a factor solution of three factors, because factor five had a small eigenvalue (1.021) and the difference between factor three and four was small (1.364 vs. 1.224). Bartlett’s test of sphericity was significant (χ² = 1358.09, df = 120, *p* < .001), KMO had middling suitability of the correlation matrix for EFA with 0.76. The three-factor solution with eigenvalue > 1.0 explained 36.11% of the total variance with the rotated loadings shown in Table 3. The first factor comprised patient anxiety, family anxiety, depression and feeling at peace. The second factor included weakness, poor appetite, sore mouth, drowsiness and poor mobility, and the third factor included sharing feelings, information and practical matters.

**Fig. 2:**
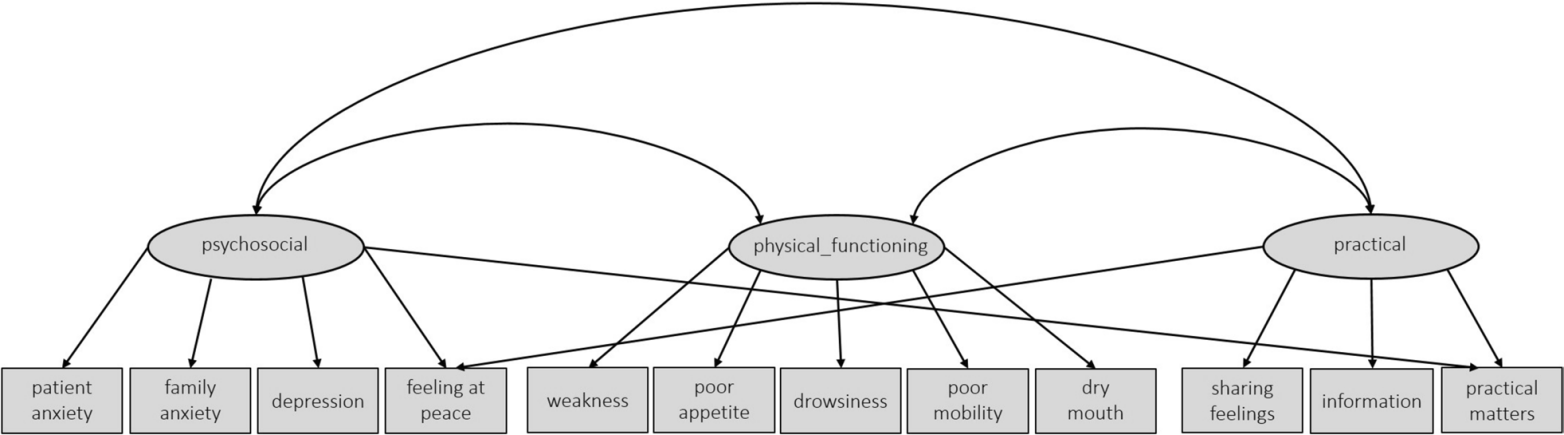
Factor model of CFA for oncological episodes

We conducted a CFA for the three-factor solution of EFA for verification. The results of CFA indicated that the designated factor model did not have a good fit (χ² = 123.079, df = 51, *p* = .000, CFI = .935, SRMR = .057, RMSEA = .07). We identified cross loadings of ‘feeling at peace’ and ‘practical matters’ and modified the factor model accordingly. The final fit indicated an adequate model fit (χ² = 90.169, df = 49, *p* = .000, CFI = .963, SRMR = .0468, RMSEA = .05). Based on this (Fig. 2), the following symptom clusters were identified: a psychosocial cluster (patient anxiety, family anxiety, depression, feeling at peace and practical matters), a physical functioning related cluster (weakness, poor appetite, drowsiness, poor mobility and dry mouth) and a communicational/practical cluster (sharing feelings, information, practical matters and feeling at peace).

### Symptom/problem clusters by diagnosis group, age and gender

In the non-oncological group, a higher percentage of patients experienced at least one symptom cluster (Table 4). The mean age was highest (84 years) in non-oncological patients with the physical cluster and lowest (68 years) in oncological patients with the psychosocial cluster. No significant gender differences were found in the distribution of symptom clusters. Median AKPS was higher in patients with the psychosocial cluster than in patients with physical cluster (30% vs. 20%) in episodes with non-oncological patients, and highest in patients with communicational/practical cluster (60%) in episodes of oncological patients. The distribution of cancer sites and type of non-oncological disease among patients with symptom clusters was similar to the whole population, with no apparent differences (Tables 1/4).

**Table 4:** Characteristics of episodes with patients with symptom/problem clusters^b^

	*Non-oncological*			*Oncological*			
	**Any** **Cluster**	**Psychosocial cluster**	**Physical cluster**	**Any** **cluster**	**Psychosocial cluster**	**Physical cluster**	**Communicational/Practical cluster**
total, *n (%)*	83/212 (39.2)	12/212 (5.7)	79/212 (37.3)	172/566 (30.4)	87/566 (15.4)	86/566 (15.2)	50/566 (8.8)
Age, years *mean ± SD*	83.8 ± 7.8	82.8 ± 11	83.6 ± 7.5	71.1 ± 12.5	67.6 ± 13.3	74.4 ± 11.2	69.3 ± 11.9
Gender, *n (%)*							
Female	47 (56.6)	8 (66.7)	45 (57.0)	80 (46.5)	40 (46.0)	42 (48.8)	24 (48.0)
Male	36 (43.4)	4 (33.3)	34 (43.0)	92 (53.5)	47 (54.0)	44 (51.2)	26 (52.0)
AKPS, *median (range)*	20 (10–50)	30 (20–50)	20 (10–50)	40 (10–90)	40 (10–90)	30 (10–80)	60 (10–90)
Diagnosis, *n (%)*							
Oncological							
Lip, mouth, pharynx				3 (1.7)	3 (3.4)	0	2 (4.0)
Gastrointestinal				51 (29.7)	27 (31.0)	26 (30.2)	14 (28.0)
Respiratory				37 (21.5)	19 (21.8)	18 (20.9)	10 (20.0)
Melanoma				1 (0.6)	1 (1.1)	0	0
Neurology				5 (2.9)	2 (2.3)	3 (3.5)	2 (4.0)
Mamma				12 (7.0)	6 (6.9)	8 (9.3)	4 (8.0)
Genitourinary				40 (23.3)	20 (23.0)	20 (23.3)	9 (18.0)
Eye, Head, CNS				1 (1.1)	1 (1.1)	0	0
Endocrinology				1 (0.6)	0	1 (1.2)	0
Others				21 (12.2)	8 (9.2)	10 (11.6)	9 (18.0)
Non-oncological							
Mental and behavioral disorders	17 (20.5)	2 (16.7)	17 (21.5)				
Neurology	10 (12.0)	1 (8.3)	10 (12.7)				
Circulatory	26 (31.3)	4 (33.3)	23 (29.1)				
Respiratory	13 (15.7)	2 (16.7)	13 (16.5)				
Gastrointestinal	10 (12.0)	2 (16.7)	9 (11.4)				
Genitourinary	6 (7.2)	0	6 (7.6)				
Others	1 (1.2)	1 (8.3)	1 (1.3)				

^b^Symptom clusters were defined as present if the IPOS values of the cluster were at least scored with a value of ‘2’ in each episode

## Discussion

We present a national study of differences in symptom burden and psychosocial problems between oncological and non-oncological patients in SPHC, and their diagnosis-related symptom and problem clusters. In addition to the symptom and problem clusters already described for the inpatient setting, this is, to our knowledge, the first attempt to examine symptom and problem clusters in patients with advanced illnesses in the home care setting in Germany. The most prevalent symptom burden was weakness and poor mobility in both groups. While the oncological patient group had a higher number of burdensome symptoms, the burden related to symptoms and problems of non-oncological patients were higher.

We identified two symptom clusters for episodes of non-oncological patients: a psychosocial cluster and a physical functioning cluster. For episodes of oncological patients, three clusters were identified: a psychosocial cluster, a physical functioning cluster, and a communicational /practical cluster. These clusters represent the dimensions of palliative care as an adequate explanatory model and include the different levels of physical symptom burden and psychosocial concerns in the unit of care and, additionally, care organization. The main difference between symptom clusters of non-oncological and oncological episodes was the existence of the communicational/practical problem cluster in the latter. This does not mean that practical problems are not prevalent or do not occur in the non-oncological episodes, however, they did not have strong correlations with other symptoms or problems that form a cluster, in our data. Oncological patients with this cluster were younger and had a higher functional status compared to all oncological episodes. Psychosocial clusters of oncological and non-oncological episodes only differed in their cross loadings: while in non-oncological episodes ‘drowsiness’ often clusters with psychosocial symptoms, in oncological episodes they occurred with practical problems. Non-oncological patients had shorter episodes of care, with a significantly higher number ending with death. We can therefore assume that they were admitted to SPHC later, as seen in other studies, [31–33] in contrast to their usual longer disease trajectories, [34] but we do not know at what point patients died. Complementary to this, Just et al. (2021) identified a significantly reduced survival time of non-oncological patients in SPHC, with performance status and age as the most important predictors of low life expectancy [31]. On average, these patients were older, and supporting services such as nursing services may have already been involved, possibly explaining the less prevalent practical matters of these patients.

Most other studies in patients with advanced cancer identified four common symptom clusters: anxiety–depression, nausea–vomiting, nausea–appetite loss, and fatigue–dyspnea-drowsiness–pain [12]. In contrast to this, as well as studies of symptom clusters of non-oncological patients [13–15], we could only identify the anxiety-depression cluster embedded in a broader psychosocial cluster. There may be different reasons, e.g. patient sample, assessment tools and statistical methods, as mentioned in Barsevick et al. (2006) [16]. While almost all identified studies focused on patients in inpatient settings or outpatient clinics, our data describe patients in the community setting, indicating high overall symptom burden and patients being close to death [17, 35]. Correspondingly, the physical symptom cluster includes mostly symptoms associated with physical decline and illness progression. In contrast to other studies [10, 25, 36–39], pain and breathlessness as leading symptoms in palliative care did not occur in the identified symptom and problem clusters, neither in the oncological nor in the non-oncological patient group. Although both symptoms are common and frequently occurring symptoms in patients with advanced disease [40], and were also prevalent in our data, they did not correlate with other symptoms in this analysis. This is possibly due to a discrepancy between clinically perceived and statistically identified symptom clusters [36].

Regarding the assessment tools, most studies calculated clusters based on assessments of symptom severity or distress [25, 38, 41, 42] e.g., using the Edmonton Symptom Assessment System [43] (ESAS). So far, no study applied proxy-reported assessment tools that address symptom burden. The methodological impact on cluster occurrence and composition should be considered [36]. Some severity assessments might not capture the whole range of symptoms that palliative care patients experience, which may result in underidentification of clusters [12]. In contrast, IPOS contains 17 items including a broad range of aspects of palliative care, and covers patients’ burden of main symptoms, family distress as well as existential, spiritual and practical concerns. This allows identification of symptom clusters that are related to family distress and practical problems and cover all dimensions of SPC [24]. Overall, our results for oncological episodes are in line with the main results of the IPOS validation study of Murtagh et al. (2019), which indicated three main subscales. Nevertheless, in contrast to Murtagh et al., our physical clusters display the symptoms that are associated with the progression of the disease and the proximity to death/dying process and not all physical symptoms. This may indicate that our patient sample differed from the sample in the validation study with regards to disease progression. Also, the communicational/practical cluster is missing in the non-oncological episodes. This may be due to the small number of non-oncological patients (15%) included in the validation study, leading to an underrepresentation of this group. Furthermore, no setting-specific differences were taken into account, which could explain specific clusters for patients in the community setting in our results [24].

Moreover, different methods for the statistical calculation of symptom clusters are used in various other studies, most commonly principal component analysis, EFA, and hierarchical cluster analysis, which tend to identify different clusters [12, 41, 44, 45]. Only anxiety and depression seem to occur within the same cluster regardless of statistical analysis [25]. In order to develop robust cluster models, a CFA is useful because it is not only based on visual evaluation of rotated factor loadings as in EFA, but on a goodness-of-fit test which quantifies how well the data actually fit the hypothesized model [46].

Family distress was identified as a relevant problem which occurs simultaneously with other psychosocial problems. Caregivers are an integral part of the unit of care but also provide most of the care at home. They take a high level of responsibility regarding practical and medical assistance, as well as emotional support. Therefore, this topic and its associated problems like patient anxiety and depression are of key importance for SPHC teams, and it is crucial that reliable, well-coordinated professional support is provided to caregivers [47].

Overall, to provide good and adequate care to people who are at the end of their life, it is necessary to understand how their symptoms might progress and to develop care plans accordingly. Especially in non-oncological patients, the trajectories are often more individual with unpredictable exacerbations, [34] and may differ from oncological patients with respect to symptom-related therapies [33]. The results demonstrate that symptom burden is perceived differently by various patient groups. SPHC teams must therefore take into account and anticipate the individual needs of patients reflected in the different manifestations of the symptom clusters. The psychosocial burden regarding both patient and family was similar in both groups, but more associated with physical issues in the non-oncological patients and with practical problems in the oncological group, which indicates the high relevance of coordination of care in a multidisciplinary SPHC team.

### Strengths and limitations

The main strength of this analysis lies in the prospectively collected national data set of oncological and non-oncological patients in SPHC using proxy assessments. Although there is evidence that some of the experienced symptoms and problems are perceived differently by patients and professionals, [24, 48] the use of proxy assessments allowed us to include all patients admitted to SPHC without exclusion due to physical decline, cognitive impairment, or inability to consent. Data reliability was maximized through SPHC staff training in application of the assessments, regular feedback, and plausibility checks during data collection, which reduced data bias and missing data.

However, the presented symptom and problem clusters have been developed based on SPHC data, hence cannot be transferred to other, especially inpatient settings, without further evaluation. Further limitations are the restriction to complete cases in the factor analyses, where not assessable items were treated like missing values. Although there were only few missing values, there were many symptoms and problems that could not be assessed, especially in non-oncological episodes and regarding psychosocial problems. For these values, we cannot reliably determine the reasons why they could not be assessed and if they occur at random, and therefore an imputation of values was not feasible. Furthermore, there were considerably more episodes from patients with oncological diseases in total. Due to the small proportion of non-oncological episodes, we did not distinguish between different diseases or disease groups. Although episodes of non-oncological patients were underrepresented, this does not impact the overall aim of achieving a better understanding of the differences between these groups in SPHC. We revealed diagnosis-related differences in the prevalence of symptom burden and psychosocial problems and the corresponding clusters, but we did not aim to explore their consistency [42] or stability [12] over time. Especially for non-oncological patients, further analysis is needed regarding their symptom and problem trajectories in SPHC. It should also be examined if symptom clusters worsen outcomes (e.g., unwanted hospitalizations, symptom relief, caregiver satisfaction) or impact the resources required in SPHC.

## Conclusions

In conclusion, we identified two clusters for non-oncological patients and three clusters for oncological patients with various correlating symptoms and problems within the clusters. They differed from other studies that assessed symptom clusters in advanced diseases, possibly due to a different patient group and different setting. The physical symptom cluster included symptoms that are related to disease progression and closeness to death. Clusters identified so far for advanced cancer do not entirely fit patients’ needs at the end of life. The main difference between groups was the identified communicational/practical cluster of oncological patients. This cluster shows the key importance of care coordination in SPHC, especially for younger cancer patients. The results indicate that symptom clusters are more prevalent in the non-oncological patient group but may respond to better symptom and problem control for both oncological as well as non-oncological patients. SPHC teams predominantly cared for cancer patients, however, non-oncological patients were more burdened by both individual symptoms and symptom clusters at the beginning of episodes, possibly indicating under-provision of SPHC for these patients. It should be examined if their earlier inclusion in SPHC can better preserve their quality of life at the end of life.

### Supplementary Information

**Additional file 1: Supplementary Table 1.** ICD-10 Diagnostic Coding Framework according to World Health Organization

**Additional file 2: Supplementary Table 2.** Frequencies of IPOS (n;%)

**Additional file 3: Supplementary Table 3.** Number of IPOS items assessed at least ‘3’ and ‘4’ (n;%)

**Additional file 4: Supplementary Figure 1.** Screeplot of exploratory factor analysis (non-oncological)

**Additional file 5: Supplementary Figure 2.** Screeplot of exploratory factor analysis (oncological)

**Additional file 6: Supplementary File 4.** COMPANION Study Group

## Data Availability

Data are available upon reasonable request.

## Abbreviations

SPHC: Specialist palliative home care
SPC: Specialist palliative care
STROBE: Strengthening the Reporting of Observational Studies in Epidemiology
STROSA: STandardized Reporting Of Secondary data Analyses
AKPS: Australia-modified Karnofsky Performance Status
IPOS: Integrated Palliative care Outcome Scale
EFA: Exploratory factor analysis
CFA: Confirmatory factor analysis
KMO: Kaiser-Meyer-Olkin
CFI: Confirmatory fit index
RMSEA: Root mean square error of approximation
SRMSR: Standardized root mean squared residual
ESAS: Edmonton Symptom Assessment System
