# Supplementary material for: Symptom and problem clusters in German specialist palliative home care - a factor analysis of non-oncological and oncological patients’ symptom burden
Source: BMC Palliat Care. 2023 Nov 17;22:183. doi: 10.1186/s12904-023-01296-0 (PMC10655459; doi:10.1186/s12904-023-01296-0)
Supplement: Supplementary file 1 — Additional file 1: Supplementary Table 1. ICD-10 Diagnostic Coding Framework according to World Health Organization [file 12904_2023_1296_MOESM1_ESM.docx]

| Supplementary Table 1. ICD-10 Diagnostic Coding Framework according to World Health Organization | |
| --- | --- |
| *Diagnostic Group* | *ICD-10 Numbers* |
| Oncology  Lip, mouth, pharynx  Gastrointestinal  Respiratory  Melanoma  Neurology  Mamma  Genitourinary  Eye, Head, CNS  Endocrinology  Others | C01 C02.1 C06.9 C07 C09.9 C10.9 C13 C13.9  C15 C15.1 C15.5 C15.8 C15.9 C16.0 C16.9 C18.0 C18.2 C18.7 C18.9 C20 C22 C22.1 C22.9 C23 C24 C25 C25.2 C25.9 C26  C32.9 C34.0 C34.1 C34.3 34.9  C43.9 C44.3 C44.5 C44.9  C45 C49.6 C49.9  C50.1 C50.9  C51.9 C52 C53.9 C54.1 C54.9 C55 C56 C61 C64 C67.6 C67.9 C68.9  C69.3 C69.9 C71.2 C71.6 C71.7 C71.9  C74  C78.6 C78.7 C79.3 C79.4 C79.88 C80 C80.9 C82.9 C83.3 C85.1 C85.9 C88 C90 C91.1 C92 C92.9 D03.4 D37.6 D43.2 D46.9 D47.4 D48 D48.2 |
| Mental and behavioral disorders | F00 F00.2 F01 F01.9 F03 F45.41 |
| Neurology | G04.9 G12.2 G20.9 G23.1 G23.3 G30.9 G31.9 G35.2 G35.9 G37.8 G40.2 G40.9 G41.9 G51.9 G54.6 G71 G81.1 D32.9 M48.06 |
| Circulatory | I11.00 I25.19 I25.5 I26.9 I34 I35 I42 I42.9 I50.01 I50.14 I50.9 I51.9 I61 I61.9 I63.3 I63.4 I63.5 I63.9 I67.88 I70 I70.24 I70.29 I71.9 I72.9  E83.5 |
| Respiratory | J18 J18.9 J44 J44.19 J44.89 J44.99 J45.9 J61 J69 J84.1 J86.9 U07.1 |
| Gastrointestinal | K56.7 K63.1 K70.3 K74.6 K76.7 K83 K92.2  D13.6 |
| Genitourinary | N17.99 N18.3 N18.4 N18.5 N19  E27.2 |
| Others | R54 L89.94 |

(https://icd.who.int/browse10/2019/en)
