# Supplementary material for: Symptom and problem clusters in German specialist palliative home care - a factor analysis of non-oncological and oncological patients’ symptom burden
Source: BMC Palliat Care. 2023 Nov 17;22:183. doi: 10.1186/s12904-023-01296-0 (PMC10655459; doi:10.1186/s12904-023-01296-0)
Supplement: Supplementary file 2 — Additional file 2: Supplementary Table 2. Frequencies of IPOS (n;%) [file 12904_2023_1296_MOESM2_ESM.docx]

Supplementary Table 2. Frequencies of IPOS (n;%)

| *Non-oncological patient group* | | | | | | | | | | | | | | | | | | | | | | | | |  |
| --- | --- | --- | --- | --- | --- | --- | --- | --- | --- | --- | --- | --- | --- | --- | --- | --- | --- | --- | --- | --- | --- | --- | --- | --- | --- |
|  | n | 0 | | | 1 | | | 2 | | | 3 | | | | 4 | | | | -99 | | | | | |  |
| pain | 212 | 37 | 17,5% | | 66 | 31,1% | | 60 | 28,3% | | 35 | | 16,5% | | 3 | | 1,4% | | 11 | | 5,2% | |  |  |  |
| shortness of breath | 210 | 72 | 34,3% | | 47 | 22,4% | | 50 | 23,8% | | 25 | | 11,9% | | 9 | | 4,3% | | 7 | | 3,3% | |  |  |  |
| weakness | 210 | 4 | 1,9% | | 11 | 5,2% | | 31 | 14,8% | | 70 | | 33,3% | | 85 | | 40,5% | | 9 | | 4,3% | |  |  |  |
| nausea | 212 | 137 | 64,6% | | 22 | 10,4% | | 19 | 9,0% | | 6 | | 2,8% | | 2 | | 0,9% | | 26 | | 12,3% | |  |  |  |
| vomiting | 212 | 173 | 81,6% | | 14 | 6,6% | | 12 | 5,7% | | 3 | | 1,4% | | 1 | | 0,5% | | 9 | | 4,2% | |  |  |  |
| poor appetite | 212 | 25 | 11,8% | | 43 | 20,3% | | 41 | 19,3% | | 34 | | 16,0% | | 43 | | 20,3% | | 26 | | 12,3% | |  |  |  |
| constipation | 212 | 91 | 42,9% | | 51 | 24,1% | | 35 | 16,5% | | 14 | | 6,6% | | 7 | | 3,3% | | 14 | | 6,6% | |  |  |  |
| dry mouth | 208 | 66 | 31,7% | | 63 | 30,3% | | 36 | 17,3% | | 26 | | 12,5% | | 8 | | 3,8% | | 9 | | 4,3% | |  |  |  |
| drowsiness | 210 | 35 | 16,7% | | 44 | 21,0% | | 55 | 26,2% | | 33 | | 15,7% | | 30 | | 14,3% | | 13 | | 6,2% | |  |  |  |
| poor mobility | 211 | 1 | 0,5% | | 9 | 4,3% | | 29 | 13,7% | | 69 | | 32,7% | | 96 | | 45,5% | | 7 | | 3,3% | |  |  |  |
| patient anxiety | 211 | 23 | 10,9% | | 35 | 16,6% | | 44 | 20,9% | | 23 | | 10,9% | | 7 | | 3,3% | | 79 | | 37,4% | |  |  |  |
| family anxiety | 211 | 4 | 1,9% | | 24 | 11,4% | | 48 | 22,7% | | 79 | | 37,4% | | 29 | | 13,7% | | 27 | | 12,8% | |  |  |  |
| depression | 207 | 20 | 9,7% | | 44 | 21,3% | | 37 | 17,9% | | 26 | | 12,6% | | 9 | | 4,3% | | 71 | | 34,3% | |  |  |  |
| feeling at peace | 209 | 9 | 4,3% | | 53 | 25,4% | | 37 | 17,7% | | 12 | | 5,7% | | 1 | | 0,5% | | 97 | | 46,4% | |  |  |  |
| sharing feelings | 206 | 9 | 4,4% | | 45 | 21,8% | | 29 | 14,1% | | 14 | | 6,8% | | 7 | | 3,4% | | 102 | | 49,5% | |  |  |  |
| information | 207 | 21 | 10,1% | | 56 | 27,1% | | 21 | 10,1% | | 4 | | 1,9% | | 8 | | 3,9% | | 97 | | 46,9% | |  |  |  |
| practical matters | 204 | 27 | 13,2% | | 75 | 36,8% | | 50 | 24,5% | | 10 | | 4,9% | | 5 | | 2,5% | | 37 | | 18,1% | |  |  |  |
| *Oncological patient group* | | | | | | | | | | | | | | | | | | | | | | | | | |
|  | n | 0 | | | 1 | | | 2 | | | | 3 | | | | 4 | | | | -99 | | | | | |
| pain | 566 | 75 | | 13.3% | 163 | | 28.8% | 197 | | 34.8% | | 106 | | 18.7% | | 21 | | 3.7% | | 4 | | 0.7% | |  |  |
| shortness of breath | 566 | 216 | | 38.2% | 182 | | 32.2% | 109 | | 19.3% | | 41 | | 7.2% | | 15 | | 2.7% | | 3 | | 0.5% | |  |  |
| weakness | 565 | 4 | | 0.7% | 38 | | 6.7% | 151 | | 26.7% | | 229 | | 40.5% | | 142 | | 25.1% | | 1 | | 0.2% | |  |  |
| nausea | 566 | 309 | | 54.6% | 129 | | 22.8% | 70 | | 12.4% | | 36 | | 6.4% | | 14 | | 2.5% | | 8 | | 1.4% | |  |  |
| vomiting | 566 | 430 | | 76.0% | 69 | | 12.2% | 32 | | 5.7% | | 20 | | 3.5% | | 10 | | 1.8% | | 5 | | 0.9% | |  |  |
| poor appetite | 566 | 76 | | 13.4% | 100 | | 17.7% | 163 | | 28.8% | | 152 | | 26.9% | | 63 | | 11.1% | | 12 | | 2.1% | |  |  |
| constipation | 565 | 236 | | 41.8% | 154 | | 27.3% | 102 | | 18.1% | | 45 | | 8.0% | | 13 | | 2.3% | | 15 | | 2.7% | |  |  |
| dry mouth | 565 | 240 | | 42.5% | 149 | | 26.4% | 118 | | 20.9% | | 37 | | 6.5% | | 15 | | 2.7% | | 6 | | 1.1% | |  |  |
| drowsiness | 563 | 115 | | 20.4% | 167 | | 29.7% | 181 | | 32.1% | | 74 | | 13.1% | | 24 | | 4.3% | | 2 | | 0.4% | |  |  |
| poor mobility | 562 | 16 | | 2.8% | 73 | | 13.0% | 178 | | 31.7% | | 178 | | 31.7% | | 116 | | 20.6% | | 1 | | 0.2% | |  |  |
| patient anxiety | 561 | 30 | | 5.3% | 129 | | 23.0% | 195 | | 34.8% | | 118 | | 21.0% | | 33 | | 5.9% | | 56 | | 10.0% | |  |  |
| family anxiety | 563 | 8 | | 1.4% | 63 | | 11.2% | 165 | | 29.3% | | 174 | | 30.9% | | 75 | | 13.3% | | 78 | | 13.9% | |  |  |
| depression | 557 | 44 | | 7.9% | 143 | | 25.7% | 179 | | 32.1% | | 116 | | 20.8% | | 26 | | 4.7% | | 49 | | 8.8% | |  |  |
| feeling at peace | 558 | 21 | | 3.8% | 219 | | 39.2% | 163 | | 29.2% | | 48 | | 8.6% | | 21 | | 3.8% | | 86 | | 15.4% | |  |  |
| sharing feelings | 558 | 44 | | 7.9% | 216 | | 38.7% | 124 | | 22.2% | | 47 | | 8.4% | | 13 | | 2.3% | | 114 | | 20.4% | |  |  |
| information | 558 | 88 | | 15.8% | 257 | | 46.1% | 88 | | 15.8% | | 33 | | 5.9% | | 2 | | 0.4% | | 90 | | 16.1% | |  |  |
| practical matters | 548 | 63 | | 11.5% | 194 | | 35.4% | 167 | | 30.5% | | 42 | | 7.7% | | 17 | | 3.1% | | 65 | | 11.9% | |  |  |
