# Supplementary material for: Symptom and problem clusters in German specialist palliative home care - a factor analysis of non-oncological and oncological patients’ symptom burden
Source: BMC Palliat Care. 2023 Nov 17;22:183. doi: 10.1186/s12904-023-01296-0 (PMC10655459; doi:10.1186/s12904-023-01296-0)
Supplement: Supplementary file 3 — Additional file 3: Supplementary Table 3. Number of IPOS items assessed at least ‘3’ and ‘4’ (n;%) [file 12904_2023_1296_MOESM3_ESM.docx]

Supplementary Table 3. Number of IPOS items assessed at least ‘3’ and ‘4’ (n;%)

|  | **Non-Oncological (n=212)** | | **Oncological (n=566)** | |
| --- | --- | --- | --- | --- |
|  | Number of severe and overwhelming symptoms and problems | | | |
| 0 symptoms/problems | 12 | 5.7% | 58 | 10.2% |
| 1-4 symptoms/problems | 114 | 53.8% | 304 | 53.7% |
| more than 5 symptoms/problems | 86 | 40.6% | 204 | 36.0% |
