# Supplementary figures and images for: Symptom and problem clusters in German specialist palliative home care - a factor analysis of non-oncological and oncological patients’ symptom burden
Source: BMC Palliat Care. 2023 Nov 17;22:183. doi: 10.1186/s12904-023-01296-0 (PMC10655459; doi:10.1186/s12904-023-01296-0)

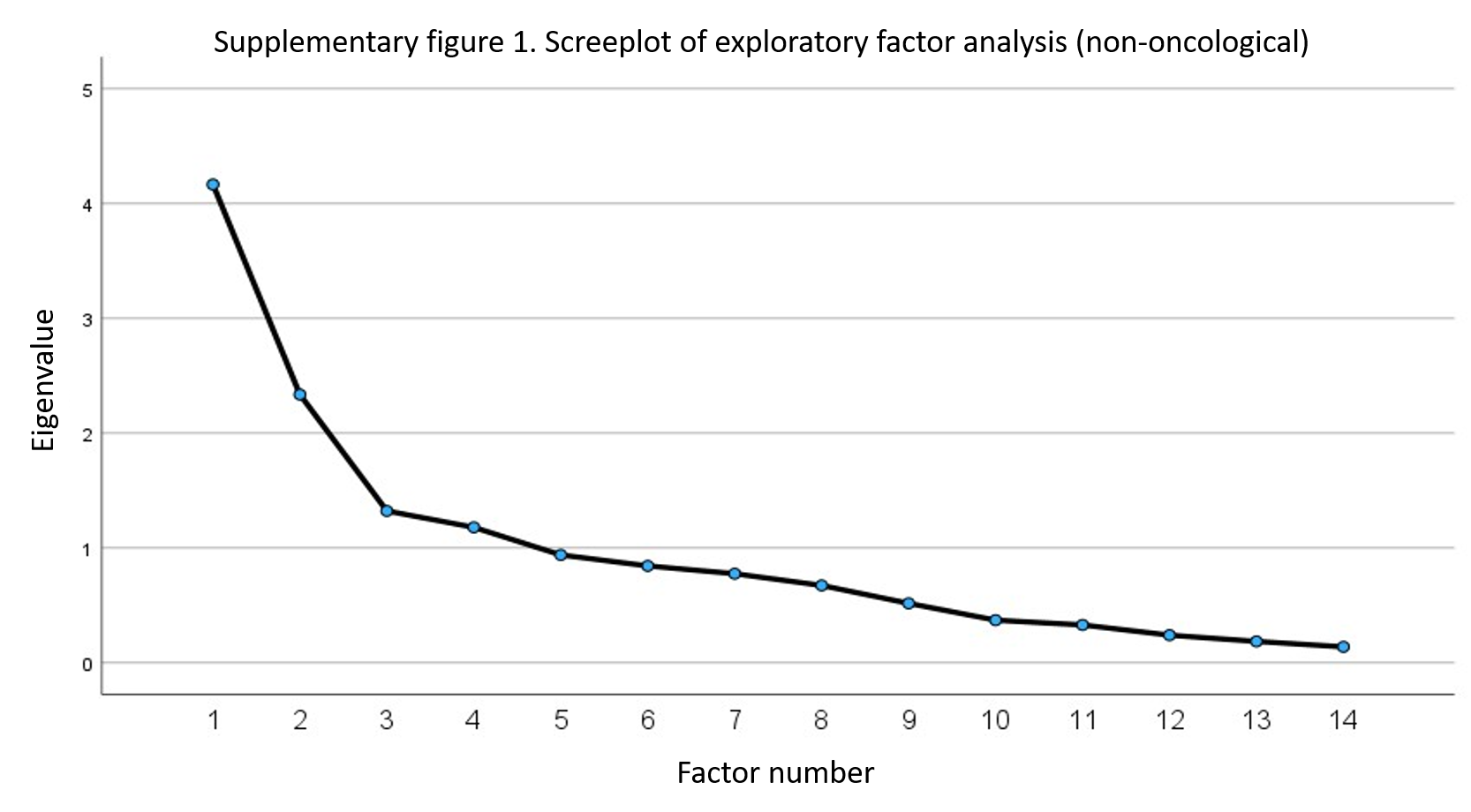

Supplement: Supplementary file 4 — Additional file 4: Supplementary Figure 1. Screeplot of exploratory factor analysis (non-oncological) [file 12904_2023_1296_MOESM4_ESM.png]

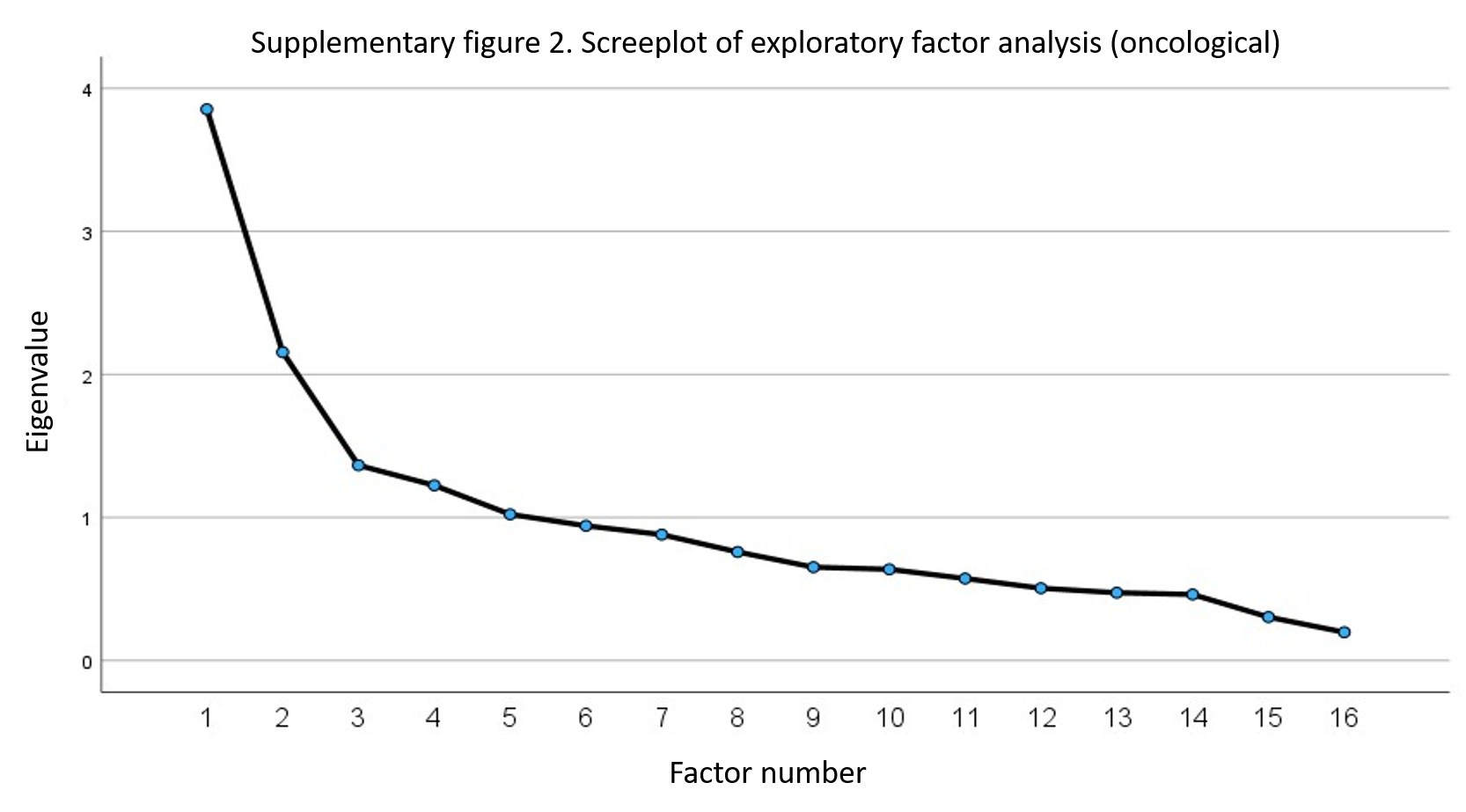

Supplement: Supplementary file 5 — Additional file 5: Supplementary Figure 2. Screeplot of exploratory factor analysis (oncological) [file 12904_2023_1296_MOESM5_ESM.png]
