# Supplementary material for: Symptom and problem clusters in German specialist palliative home care - a factor analysis of non-oncological and oncological patients’ symptom burden
Source: BMC Palliat Care. 2023 Nov 17;22:183. doi: 10.1186/s12904-023-01296-0 (PMC10655459; doi:10.1186/s12904-023-01296-0)
Supplement: Supplementary file 6 — Additional file 6: Supplementary File 4. COMPANION Study Group [file 12904_2023_1296_MOESM6_ESM.docx]

**COMPANION Study Group**

Farina Hodiamont^1^, Claudia Bausewein^1^, Julia Wikert^1^, Eva Lehmann-Emele^1,2^, Daniela Gesell^1^, Maximiliane Jansky^2^, Friedemann Nauck^2^, Christina Niessl^3^, Theresa Ullmann^3^, Anne-Laure Boulesteix^3^, Caroline Schatz^4,5^, Reiner Leidl^4,5^, Alisa Stöber^5^, Katharina Schoder^5^, Renee Stark^5^, Steven Kranz^6^, Heiner Melching^6^

^1^ Department of Palliative Medicine, LMU University Hospital, LMU Munich, Germany.

^2^ Department of Palliative Medicine, University Medical Center Goettingen, Goettingen, Germany.

^3^ Institute for Medical Information Processing, Biometry, and Epidemiology (IBE), Ludwig-Maximilians-University Munich (LMU), Munich, Germany.

^4^ Ludwig-Maximilians-Universität München, LMU Munich School of Management, Institute of Health Economics and Health Care Management, Munich, Germany.

^5^ Helmholtz Zentrum München, Institute of Health Economics and Health Care Management, Munich, Germany.

^6^ German Association for Palliative Medicine, Berlin, Germany.
